# Role of tear vasoactive intestinal peptide on dry eyes after laser keratorefractive surgery

**DOI:** 10.1186/s12886-023-02857-w

**Published:** 2023-04-20

**Authors:** Yang Kang, Qi Hu, Xue Li, Zidan Guo, Qiong Wu, Hong Zhang

**Affiliations:** 1 Eye Hospital, The First Affiliated Hospital of Harbin Medical University, No. 143, Yiman Street, 150001 Harbin, Heilongjiang Province People’s Republic of China; 2 Outpatient department of Harbin Medical University, Harbin Medical University, Harbin, Heilongjiang Province People’s Republic of China

**Keywords:** Dry eye, FS-LASIK, LASEK, Vasoactive intestinal peptide, Corneal ablation depth

## Abstract

**Background:**

To explore the changes in vasoactive intestinal peptide (VIP) concentration in tears post laser-assisted sub-epithelial keratomileusis (LASEK) and femtosecond laser-assisted in situ keratomileusis (FS-LASIK) surgeries and related factors, possible association between postoperative dry eye symptoms and VIP concentration in tears, and factors influencing dry eye symptoms after different periods post LASEK and FS-LASIK surgeries.

**Methods:**

In this prospective, non-randomized, longitudinal cohort study, 23 and 22 subjects were recruited and underwent LASEK and FS-LASIK, respectively. After conducting an intact ophthalmic examination and collecting relevant surgical data, all subjects were examined for VIP concentration in their tears using ELISAs, tear-film breakup time, ocular staining and ocular surface disease index questionnaire before surgery and 1 day, 1 week, and 1 month post-surgery.

**Results:**

Tear VIP concentration increased significantly after both LASEK and FS-LASIK, with the highest concentration observed 1 week post-surgery (*P* ≤ 0.05). Tear VIP concentration correlated negatively with corneal ablation depth (AD). The extent of dry eyes was related to the operation method employed and postoperative recovery period. In FS-LASIK and LASEK subjects, dry eyes were mainly affected by the basic ocular surface status before surgery, and VIP concentration. Furthermore, in LASEK subjects, dry eyes were negatively correlated with AD.

**Conclusion:**

VIP was stimulated and mobilized as an emergency protection post-refractive surgery and a trauma model affected by AD. It can indirectly indicate the inevitable relationship between postoperative dry eye and nerve injury. Elevated post-surgery tear VIP relieves dry eye symptoms, showing its neuroimmune role in regulating adverse injury stimulation. The present study provides a solution to the pathogenesis of postoperative dry eyes.

**Trial registration:**

The trial registration number: 2021JS22. Date of registration: 10 May 2021.

## Background

Transient dry eye symptoms after laser keratorefractive surgery are common, and up to 95% of patients may have dry eyes at some time post laser-assisted in situ keratomileusis (LASIK) surgery [1]. Dry eye may lead to delayed corneal healing and unstable vision [2] in early stages. Patients with chronic dry eye may experience refractive degeneration and decreased vision quality [3]. Based on large statistics sample, factors contributing to the risk of dry eyes include pre-existing dry eye disease (DED) [4], being of Asian heritage [5], being female, age [6], diabetes [7] and high myopia [8]. However, the pathogenesis of dry eyes after laser keratorefractive surgery remains unclear, requiring further investigation.

Laser-assisted sub-epithelial keratomileusis (LASEK) and femtosecond laser-assisted in situ keratomileusis (FS-LASIK) are known to contribute to corneal nerve damage and dysfunction [9]. This could lead to post operative dry eyes condition, which is also known as neuropathic dry eye. However, neuromorphology recovery time is not synchronized with dry eye relief time [10], indicating that it could not directly reflect the degree of dry eye. Through recent research [11] speculated that the degree of dry eye may be related to neuroimmune protection mechanisms triggered by surgery and the difference in release of tear neuropeptides. The function of tear neuropeptides has been studied in ocular immune privilege, infection, DED, and allergic eye disease [12]. Certain neuropeptides are known to be expressed on the ocular surface and involved in maintaining the stability of the lacrimal gland functional unit and corneal environment [13, 14]. Vasoactive intestinal peptide (VIP) is the paradigm of an endogenous neuropeptide produced by neurons and endocrine and immune cells, involved in the control of both innate and adaptive immune responses. In addition, VIP is entitled “very important peptide” among the four tear neuropeptides. It is mainly expressed in human lacrimal gland interstitium and shows an immunoreactive function at the epithelial-stromal junction adjacent to conjunctival goblet cells [15, 16], which may play an important role in the mediation of dry eye after refractive surgery.

In this study, VIP concentration, and the factors affecting VIP secretion in the tears of patients who underwent two surgical procedures were assessed. The factors influencing dry eye at different times post LASEK and FS-LASIK surgeries, and a possible correlation between dry eye and VIP concentration were analyzed. The purpose of this study is to explore the neural mechanisms contributing to dry eyes and provide a basis for future treatment strategies.

## Methods

### Study design

A 6-month prospective, non-randomized, longitudinal cohort study was conducted at the Refractive Surgery Center of the Eye Hospital at the First Affiliated Hospital of Harbin Medical University. The study was conducted in accordance with the tenets of the Declaration of Helsinki and approved by the First Affiliated Hospital of Harbin Medical University Ethics Committee. Dry eye indices and VIP were measured before surgery and 1 day, 1 week, and 1 month post-surgery and recorded as 1, 2, 3, and 4 in chronological order. Some data were not measured 1 day after surgery to minimize the risk of damage to operated eyes, which could not affect statistical results. The severity of dry eye and increase in VIP concentration were recorded as 3 − 1, 1–3, 4 − 1, and 1–4, which indicates the difference between the values at two time points; 1 represents pre-operation, 3 represents 1 week post operation, and 4 represents 1 month post operation.

### Participants

The subjects underwent either LASEK or FS-LASIK surgery; the choice of surgery was determined by the desire of the subject based on surgical indications. The inclusion criteria for surgery were as follows: (1) subjects were 18–45 years old; (2) has a stable refractive error for at least 2 years ( < − 0.25 D change per year); (3) the estimated residual stromal bed thickness was not less than 280 µ m; and (4) the subjects with more than a -5.00D spherical equivalent only chose FS-LASIK surgery. The exclusion criteria included topography consistent with keratoconus, eyes with ocular comorbidities, previous ophthalmic operation, relevant systemic disorders, medication history, especially growth inhibitors and glucocorticoids, pregnancy, monovision, and missing medical charts.

### FS-LASIK and LASEK procedure

Standard LASEK and FS-LASIK surgical techniques were performed for all subjects by the same experienced surgeon. In LASEK, a corneal epithelial flap was created with 20% alcohol solution instilled inside the trephine centered on the pupil, leaving a superior hinge. The ablation was performed with EX500 excimer laser (Alcon Laboratories Inc., Fort Worth, TX) over an optical diameter ranging from 6.25 to 6.75 mm, surrounded by a 1.5-mm transition zone. None of the subjects were treated with mitomycin C, and a bandage contact lens (ACUVE OASYS, Johnson & Johnson, Inc.) was inserted for 7 days. In FS-LASIK, a flap with 110 μm depth, 8.5– 9.1 mm diameter, and a superior gas canal 105° in the right eye and 75° in the left eye was created using the Allegretto Wave-Light FS-200 laser (Alcon Laboratories Inc., Fort Worth, TX). Other parameters about flap creation were as follows: side-cut angle was 90°; hinge angle was 50°; and canal width was 1.5 mm in all subjects. The ablation was performed with a repetition rate of 500 kHz and pulse energy of approximately 160nJ, similar to LASEK.

### Perioperative management

Detailed systemic and ocular disease history was inquired for all subjects, followed by a complete ophthalmic examination. Relevant data were recorded in detail and the risk of surgery was comprehensively evaluated before the surgery. A standard postoperative topical steroid (Fluorometholone 0.1%; Santen Pharmaceutical Co., Ltd.) tapered 4 months for LASEK and over 12 days for FS-LASIK, topical antibiotic (Tobramycin 0.003%; Alcon Laboratories, Inc.) for 7 days; and topical lubricant eye drops (HYCOSAN, GmbH, Germany) for approximately 3 months were administered. Follow-up examinations involved measurements of uncorrected distance visual acuity, slit-lamp examination, intraocular pressure (IOP) and corneal topography at days 1 and 7, and months 1, 3 and 6 after surgery. All perioperative examinations were standardized [17, 18] without errors by the same surgical team. The medication, dosage and subsequent examinations were completely consistent throughout the study, and the subjects with changed dosages were excluded from the study.

### Outcome measures

Pooled basal tears from the eye of the subject without previous topical anesthesia were collected by inserting dry sharp-tip microsponges (Alcon Lab. Inc., Fort Worth, TX) into the inferior conjunctival fornix for 30 s, followed by storage at − 80 °C for further analysis [19]. The tear samples were diluted with 200 µl phosphate-buffered saline on ice for 30 min before centrifuging at 400 rpm for 5 min at 4 °C. Protein concentration was then determined using the BCA Protein Assay Kit (Beyotime Institute of Biotechnology). A specific enzyme-linked immunosorbent assay (ELISA) was performed to quantify the neuropeptides according to the manufacturer’s protocols after a 1:50 dilution of tear samples with sterile phosphate-buffered saline solution. (detection limit of VIP ELISA kit from Cayman Chemical Company, Ann Arbor, MI, USA: 4.69 ng/ml of VIP) [20].

The ocular surface assessment comprised tear-film breakup time (TBUT), ocular staining, and ocular surface disease index (OSDI). TBUT and corneal staining were observed using a slit- lamp microscope with a cobalt filter after corneal fluorescein staining. The test result is the time from a complete blink until the first signs of a break in the tear film, which was repeated thrice and averaged. Corneal staining was performed using the Oxford grading scale as reference [21]. The Chinese versions of the OSDI (Allergan Inc., Irvine, CA, USA) were used to quantitatively assess subjective ocular surface conditions. The thinnest corneal thickness was measured thrice using Pentacam (Oculus GmbH, Wetzlar, Germany) with quality factor > 95% under the quality specification window, and the minimum value was obtained and reported before surgery. Corneal ablation depth (AD) and predicted residual corneal thickness (RCT) were determined using the software present in the Allegretto EX500 excimer laser generated according to the actual conditions of surgery.

### Statistical analysis

All statistical analyses were performed using the SPSS software (version 21.0; SPSS Inc. Chicago, IL). Continuous variables were expressed as mean ± SD or median (interquartile range [IQR]) values and tested for normal distribution. The basic surgical parameters of subjects between the two groups were analyzed using an independent sample *t*-test. The changes in tear VIP concentration and dry eye index during the perioperative period were analyzed using nonparametric tests. The differences in VIP concentrations and dry eye indices between the two groups were simultaneously compared using the Mann Whitney-U test. VIP concentration and dry eye indices correlation analyses were examined using Pearson correlation and Spearman tests for normally and non-normally distributed data, respectively. Coefficients were used to rank dry eye factor correlation. Statistical significance was assigned for *P* ≤ 0.05.

## Results

### Participant demographics

Twenty-three subjects (23 eyes, 12 males and 11 females) for LASEK and 22 subjects (22 eyes, 10 males and 12 females) for FS-LASIK fulfilled the inclusion criteria for the study. The basic surgical parameters of these subjects are shown in Table 1. The spherical equivalent (SE), AD, and RCT of the two groups of patients were different (*p* ≤ 0.05) due to the limitation that subjects with more than − 5.00D only choose FS-LASIK.

**Table 1:** Clinical characteristics related to surgical modality in subjects

Characteristics	Subjects, No. (%)		
	Subjects with LASEK	Subjects with FS-LASIK	*P-*Value
	(n = 23)	(n = 22)	
Age (y)	24.35 ± 3.50	25.91 ± 4.23	0.258
IOP (mmHg)	14.83(13.72–15.94)	16.20(14.95–17.55)	0.091
CCT (µm)	539.30(528.30-550.30)	551.55(535.69-567.41)	0.091
AD (µm)	47.87(44.57–51.17)	83.55(77.06–90.03)	< 0.001*
RCT (µm)	441.35(429.65-453.05)	362.64(346.60-378.67)	< 0.001*
SE (D)	2.82 ± 0.50	5.95 ± 1.50	< 0.001*
Haze	0.14(0.04–0.24)	0	0.005*

IOP: intraocular pressure; CCT: central corneal thickness; AD: corneal ablation depth; RCT: residual corneal thickness; SE: spherical equivalent; D: diopters;*: *p* ≤ 0.05

### Trends in tear VIP concentrations and dry eye indices during the perioperative period

**Fig. 1:**
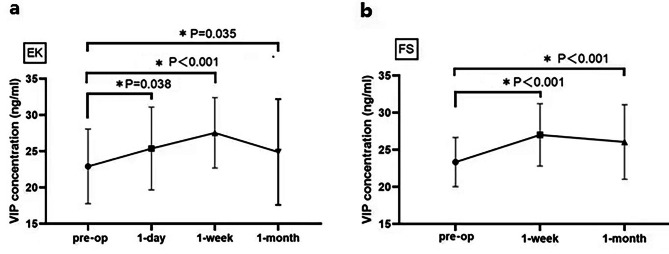
Tear VIP concentrations over time, pre- and post-surgery (**a**) Graph showing results for subjects in the LASEK (*EK)* group. (**b**) Graph showing results for subjects in the FS-LASIK (*FS)* group (*preop: preoperative, the x-axis shows the duration post-surgery).* Tears were only collected from LASEK subjects 1 day post surgery to minimize the risk of damage to operated eyes

**Fig. 2:**
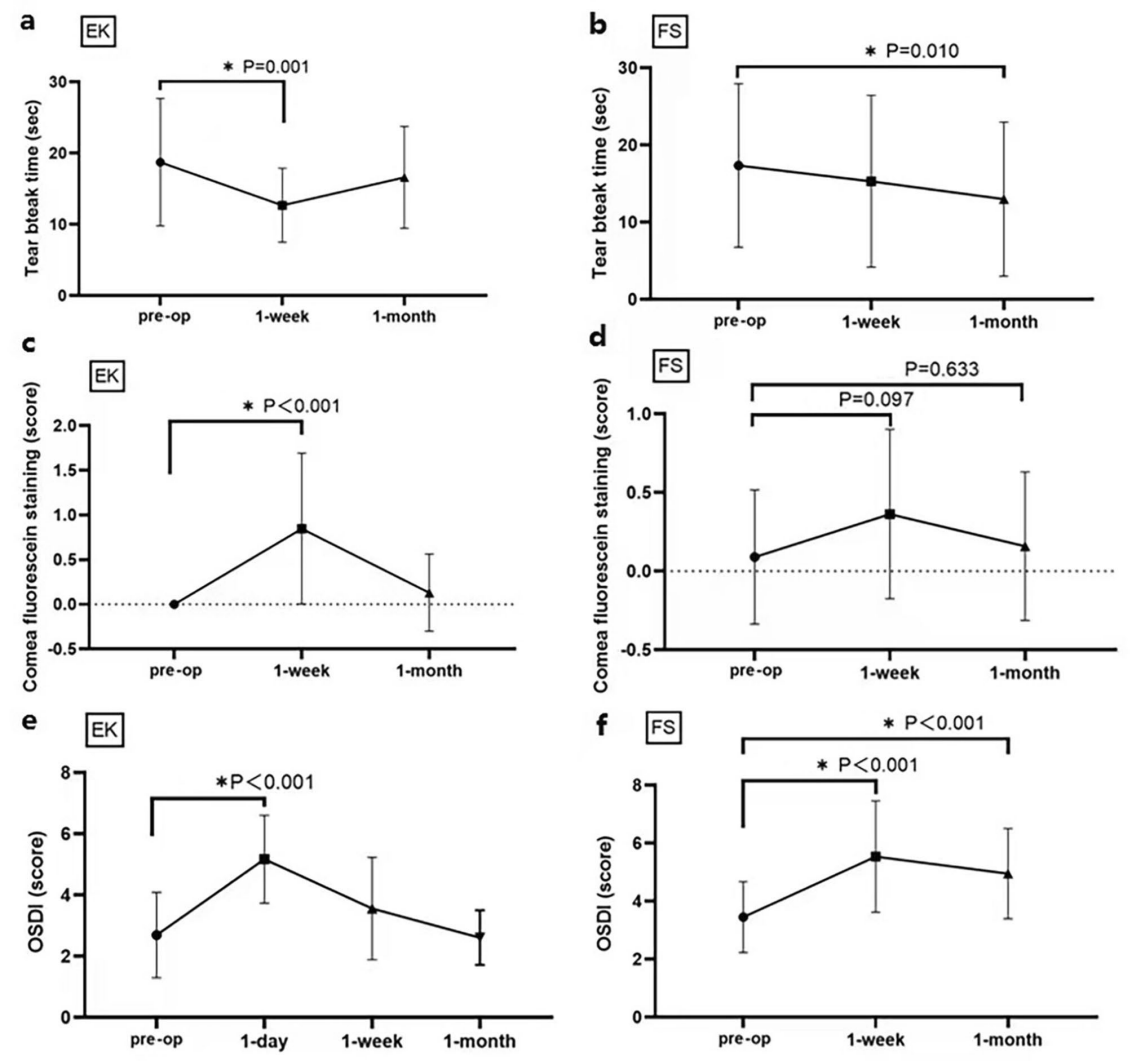
Tear-film breakup time, corneal staining, and ocular surface disease index over time pre- and post-surgery Graphs in the left-hand column (**a**, **c**, **e**) show data for LASEK (*EK)* group, while those in the right-hand column show data for FS-LASIK (*FS)* group *(preop: preoperative, the x-axis shows the duration post-surgery).* Only subjects in LASEK have OSDI on the first day after surgery, and patients in FS-LASIK were absent

Tear VIP concentration increased significantly after surgery, with the highest concentration after 1 week (*p* ≤ 0.05, Fig. 1). In the LASEK group, TBUT decreased and, corneal staining and OSDI significantly increased after 1 week, and 1 day of surgery, respectively (*p* ≤ 0.05, Fig. 2a, c, e). In the FS-LASIK group, TBUT decreased significantly after 1 month, and OSDI increased significantly after 1 week and 1 month post-surgery (*p* ≤ 0.05, Fig. 2b, d, f).

### Comparison of tear VIP concentrations and dry eye indices between the two groups

There was insignificant difference in tear VIP concentrations between the two groups at any point in time. Although the difference was insignificant, subjects in the LASEK group had more severe dry eye indices (Table 2) and a greater degree of deterioration (Table 3) than those in the FS-LASIK group after 1 week of surgery, with corneal staining showing significant differences (*P ≤* 0.05). Dry eye symptoms (Table 3) were more severe in the FS-LASIK group than in the LASEK group 1 month post-surgery, but the difference was insignificant. The OSDI in the FS-LASIK group was significantly higher than that in the LASEK group 1 week and 1 month post-surgery (*P ≤* 0.05, Tables 2 and 3).

**Table 2:** Comparison of VIP concentrations and dry eye indices between LASEK and FS-LASIK groups at different time points

Variable	Pre-op			Post-op 1-week			Post-op 1-month		
	LASEK	FS-LASIK	*P*-value	LASEK	FS-LASIK	*P*-value	LASEK	FS-LASIK	*P*-value
VIP (ng/ml)	22.93 ± 5.14	23.36 ± 3.32	0.229	27.56 ± 4.86	27.02 ± 4.20	0.982	24.91 ± 7.30	26.07 ± 5.02	0.173
TBUT (s)	18.74 ± 8.96	17.30 ± 10.60	0.424	12.70 ± 5.18	15.30 ± 11.14	0.909	16.61 ± 7.15	13.00 ± 9.98	0.036*
Ocular staining	0	0.09 ± 0.43	0.307	0.85 ± 0.85	0.36 ± 0.54	0.029*	0.13 ± 0.43	0.34 ± 0.39	0.010*
OSDI	2.70 ± 1.40	3.45 ± 1.22	0.050	3.57 ± 1.67	5.55 ± 1.92	<0.001*	2.61 ± 0.89	4.95 ± 1.56	<0.001*

Pre-op: preoperation; Post-op 1-week: Post-operative 1-week; Post-op 1-month: Post-operative 1-month; VIP: Vasoactive intestinal peptide; TBUT: Tear-film breakup time; OSDI: ocular surface disease index ; s: second; *: *p* ≤ 0.05

**Table 3:** Comparison of the aggravated degree of dry eye indices between LASEK and FS-LASIK groups post-surgery

Variable	Subjects No. (%)		*P*-Value
	LASEK	FS-LASIK	
TBUT 1–3 (s)	6.04 ± 7.81	2.05 ± 6.28	0.059
TBUT 1–4 (s)	2.13 ± 5.37	4.36 ± 7.27	0.246
Ocular staining1-3	-0.85 ± 0.85	-0.27 ± 0.74	0.024*
Ocular staining1-4	-0.13 ± 0.43	-0.25 ± 0.55	0.048*
OSDI 1–3	-0.87 ± 2.32	-2.09 ± 1.85	0.038*
OSDI 1–4	0.09 ± 1.13	-1.50 ± 1.56	<0.001*

VIP: Vasoactive intestinal peptide; TBUT: Tear-film breakup time; OSDI: ocular surface disease index; s: second; 1–3: Difference between preoperative and postoperative 1 week;1–4: Difference between preoperative and postoperative 1 month; *: *P ≤ 0.05*

### Correlation analysis of the post-surgical increase in tear VIP concentrations

**Fig. 3:**
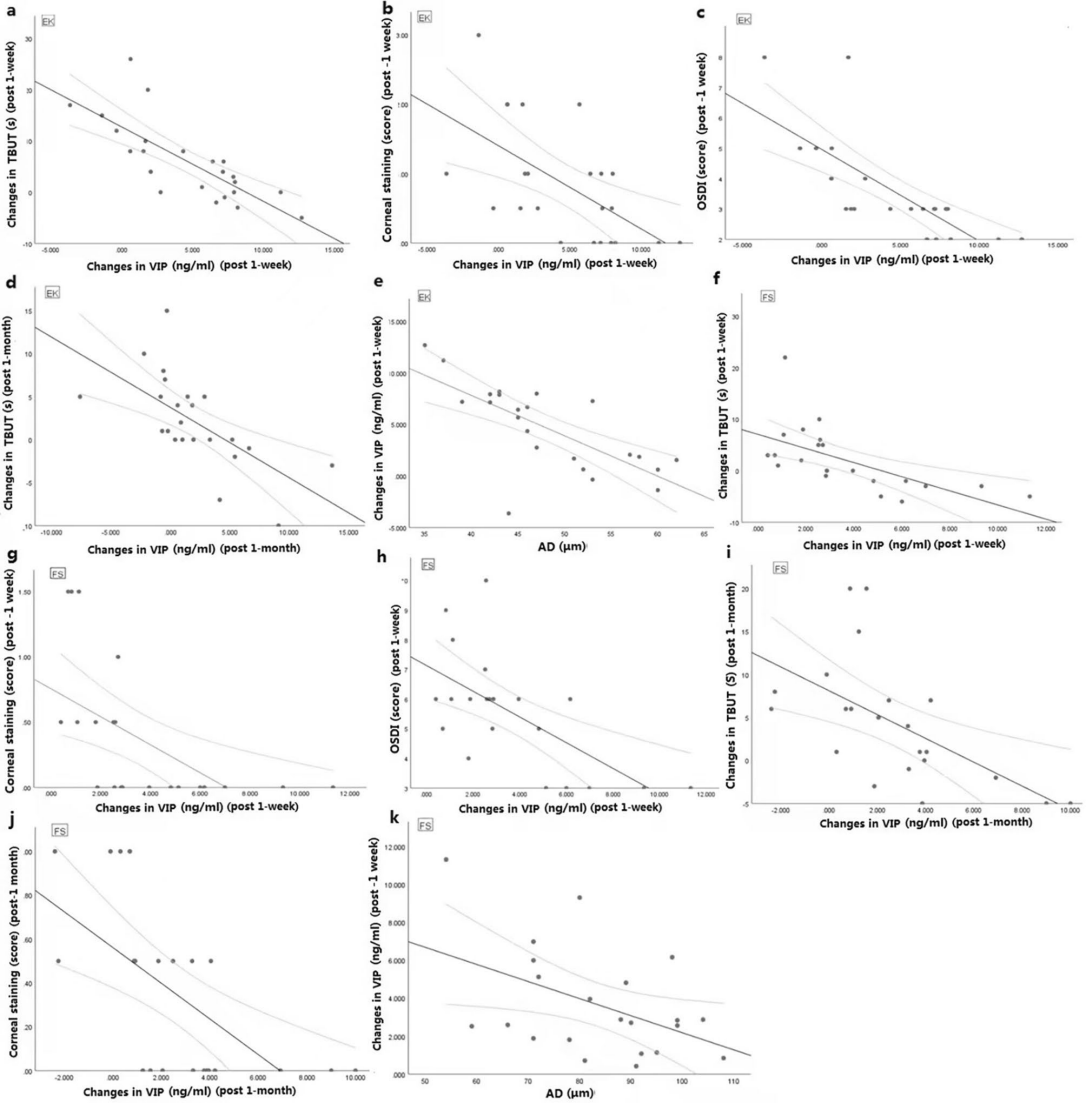
Correlation analysis between VIP concentration increase, dry eye symptom severity, and corneal AD post-surgery Graphs a-e are LASEK (EK) group, showing the correlation between VIP concentration increase (VIP3-1) and TBUT severity (TBUT1-3); (**a**), corneal staining 3 (**b**), OSDI3 (**c**), intraoperative corneal ablation (**e**) 1 week post surgery, as well as the correlation between VIP concentration increase (VIP4-1) and TBUT severity (TBUT4-1) (**d**) 1 month post surgery. Graphs f–k are FS-LASIK (FS) group, showing the correlation between VIP concentration increase (VIP3-1) and TBUT severity (TBUT1-3) (**f**), corneal staining 3 (**g**), OSDI3 (**h**), intraoperative corneal ablation (**k**) 1 week post surgery, as well as the correlation between VIP concentration increase (VIP 4 − 1) and TBUT severity (TBUT 4 − 1) (**i**), corneal staining 4 (**j**), 1 month post-surgery. There was no correlation analysis of any data one day post-surgery

Increases in VIP concentrations were correlated with dry eye indices and AD. In the LASEK group, TBUT1-3, corneal staining3, and OSDI3 were negatively correlated with VIP3-1 (R: -0.773, *P*<0.001; R:-0.591, *P* = 0.003; R: -0.821, *P*<0.001; Fig. 3a–c). TBUT1-4 levels were negatively correlated with VIP4-1 (R: -0.642, *P* = 0.001; Fig. 3d). Similarly, in the FS-LASIK group, TBUT1-3, corneal staining3, and OSDI3 were negatively correlated with VIP3-1 (R: -0.623, *P* = 0.002; R: -0.569, *P* = 0.006; R: -0.645, *P* = 0.001; Fig. 3f–h). TBUT1-4 and corneal staining 4 were negatively correlated with VIP4-1 (R: -0.587, *P* = 0.004; R: -0.643, *P* = 0.001; Fig. 3i–j). VIP3-1 was negatively correlated with AD in both the LASEK (R: -0.719, *P*<0.001; Fig. 3e) and FS-LASIK groups (R: -0.463, *P* = 0.03; Fig. 3k). No other relevant factors affecting tear VIP concentration were found, including age, IOP at each stage, central corneal thickness (CCT), RCT, and haze. Furthermore, no correlation was found between preoperative tear VIP concentration and dry eye symptoms before surgery.

### Correlation analysis of dry eye symptoms after surgery

Table 4 lists the factors associated with dry eye symptoms 1 week and 1 month post-surgery. They include TBUT1, VIP1, VIP3-1, VIP4-1, VIP3, VIP4, and AD and were sorted by their correlations coefficients. The main results are as follows: dry eye symptoms were negatively correlated with tear VIP concentration, including instant VIP and increase of VIP concentrations. The correlation was greater in the FS-LASIK group than in the LASEK group, after 1 week than 1 month post-surgery. Dry eye symptoms correlated positively with TBUT1 levels in both groups. Furthermore, dry eye symptoms were positively correlated with AD in the LASEK group.

**Table 4:** Correlation analysis of dry eye symptoms after LASEK and FS-LASIK surgeries after 1 week and 1 month, and the magnitude of the correlation compared with the coefficient

Variables ( Post-op1-week)	LASEK						FS-LASIK						
	VIP1	VIP 3	VIP 3 − 1	AD	TBUT1	VIP 1		VIP 3	VIP 3 − 1	AD	TBUT1		
TBUT1-3 ( s )	④R:0.513 *P* = 0.012^*^	R:-0.119 *P* = 0.590	②R:-0.773 *P* < 0.001^*^	③R:0.518 *P* = 0.011^*^	①R:0.817 *P* < 0.001^*^	R:-0.321 *P* = 0.145		①R:-0.676 *P* = 0.001^*^	②R:-0.623 *P* = 0.002^*^	R:0.245 *P* = 0.273	R:0.209 *P* = 0.351		
TBUT3 ( s )	R:0.227 *P* = 0.297	R:0.221 *P* = 0.311	R:-0.023 *P* = 0.918	R:-0.179 *P* = 0.415	①R:0.496 *P* = 0.016^*^	R:0.017 *P* = 0.939		③R:0.483 *P* = 0.023^*^	②R:0.692 *P* < 0.001^*^	R:-0.347 *P* = 0.113	①R:0.834 *P* < 0.001^*^		
Ocular staining3	R:-0.013 *P* = 0.954	③R:-0.520 *P* = 0.011^*^	①R:-0.591 *P* = 0.003^*^	②R:0.536 *P* = 0.008^*^	④R:-0.427 *P* = 0.042^*^	R:-0.228 *P* = 0.307		②R:-0.566 *P* = 0.006^*^	①R:-0.569 *P* = 0.006^*^	R:0.246 *P* = 0.271	R:-0.099 *P* = 0.660		
OSDI	④R:0.480 *P* = 0.020^*^	R:-0.133 *P* = 0.545	①R:-0.821 *P* < 0.001^*^	③R:0.536 *P* = 0.008^*^	②R:0.796 *P* < 0.001^*^	R:-0.210 *P* = 0.348		②R:-0.603 *P* = 0.003^*^	①R:-0.645 *P* = 0.001^*^	③R:0.569 *P* = 0.006^*^	R: 0.036 *P* = 0.874		
**Variables** **( Post-op1-month)**	**LASEK**						**FS-LASIK**						
	**VIP1**	**VIP 4**	**VIP4-1**	**AD**	**TBUT1**	**VIP 1**		**VIP4**	**VIP4-1**	**AD**	**TBUT1**		
TBUT 1–4 ( s )	R:0.163 *P* = 0.457	R:-0.256 *P* = 0.239	①R:-0.642 *P* = 0.001^*^	R:0.401 *P* = 0.058^*^	②R:0.602 *P* = 0.002^*^	③R:-0.472 *P* = 0.026^*^		①R:-0.671 *P* = 0.001^*^	②R:-0.587 *P* = 0.004^*^	R:0.019 *P* = 0.933	④R:0.426 *P* = 0.048^*^		
TBUT 4 ( s )	②R:0.602 *P* = 0.002^*^	③R:0.589 *P* = 0.003^*^	④R:-0.430 *P* = 0.041^*^	R:0.136 *P* = 0.536	①R:0.800 *P* < 0.001^*^	R:0.161 *P* = 0.474		③R:0.567 *P* = 0.006^*^	①R:0.753 *P* < 0.001^*^	R:-0.248 *P* = 0.267	②R:0.752 *P* < 0.001^*^		
Ocular staining4	R:-0.289 *P* = 0.181	R:-0.259 *P* = 0.232	R:-0.097 *P* = 0.659	R:0.112 *P* = 0.611	R:0.133 *P* = 0.547	R:-0.060 *P* = 0.791		②R:-0.432 *P* = 0.044^*^	①R:-0.643 *P* = 0.001^*^	R:0.312 *P* = 0.157	R:-0.360 *P* = 0.100		
OSDI 4	R:-0.177 *P* = 0.418	R:-0.103 *P* = 0.641	R:0.038 *P* = 0.863	R:-0.093 *P* = 0.674	R:-0.315 *P* = 0.143	R:-0.256 *P* = 0.250		R:-0.393 *P* = 0.070	R:-0.367 *P* = 0.093	①R:0.476 *P* = 0.025^*^	R:-0.109 *P* = 0.631		

Table 4 Post-op 1-week: Post-operative 1-week; Post-op 1-month: Post-operative 1-month; VIP: Vasoactive intestinal peptide; TBUT: Tear-film breakup time; OSDI: ocular surface disease index; s: second; 1–3: Difference between preoperative and postoperative 1 week;1–4: Difference between preoperative and post-operative 1 month; AD: corneal ablation depth. ①②③④⑤: magnitude of correlation; *:*p*<0.05

## Discussion

By taking refractive surgery as a trauma model in our study, this study established that postoperative stress response resulted in postoperative VIP concentration increase, which is highly related to corneal ablation during surgery. Elevated tear VIP after surgery could relieve dry eye symptoms and dry eye symptoms at different stages after surgery are highly correlated with VIP concentration and preoperative basic ocular surface status. The present study can indicate that there is a direct relationship between dry eye and nerve damage after refractive surgery. Under adverse injury stimulation, VIP plays a neuroimmune role in regulating dry eyes, including after refractive surgery.

The expression, trophic, and immune roles of VIP present in tears are the focus of this research. VIP is secreted by nerve fibers derived from the parasympathetic nerves, which are distributed mainly in corneal stroma [22], and endocrine and immune cells [23]. Once released, the 28-amino acid long VIP can bind to three types of G protein-coupled plasma membrane receptors: VPAC1, VPAC2, and PAC1 (PACAP-preferring receptor 1) [24] and is involved in a dynamic process called “neurogenic inflammation” at the ocular surface. VIP controls exocrine secretions of water and electrolytes as well as MUC5AC [25]. Therefore, this study hypothesized that VIP, a multifactorial inflammatory factor, may regulate postoperative dry eyes.

Before surgery, there was no difference between tear VIP concentration and conditions such as dry eye symptoms, IOP, age, CCT and RCT, as well as VIP concentration between the two groups. This was similar to the results of a previous study [26], in which there was no difference in tear VIP concentrations between DED and healthy control population. The increase in postoperative VIP concentration reflects a stress response to surgery, and a series of injury repairs caused by corneal nerve stimulation. Mechanical stimulation due to surgery can activate corneal or conjunctival afferent sensory nerves and stimulate parasympathetic efferent nerves [13]. This leads to the release of VIP, a parasympathetic neurotransmitter, together with acetylcholine which then triggers an immune response. The objective is to resist disturbances in lacrimal gland functional unit, neutralize environmental threats, relieve dry eye symptoms and promote ocular surface repair.

The research found that the increase in tear VIP concentration 1 week after surgery was negatively correlated with corneal AD in both LASEK and FS-LASIK groups: that is, the greater the AD, the lower the increase in VIP concentration. Ablation of the anterior corneal stroma aimed at changing the refraction of myopia could injure the parasympathetic nerves present in it [11], inhibit the release of VIP, and lead to an elevated difference in tear VIP concentration after both surgeries. Therefore, the results confirmed the nerve fiber factors affecting VIP release. The increase in VIP concentration post surgery is affected by corneal ablation, which is determined by the degree of myopia before surgery.

This study found a significant correlation between VIP concentration and dry eye symptoms, that is, the increase in VIP under stress plays a protective role in relieving dry eye symptoms after corneal injury. This role can be explained by considering the following two aspects: VIP shows an immunoreactive function along the epithelial-stromal junction adjacent to goblet cells, where it stimulates MUC5AC secretion by conjunctival goblet cells [16]. In addition, it controls exocrine secretion of the lacrimal gland cells including acinar, duct, and myoepithelial (MECs) cells through its expression in the human lacrimal gland interstitium [27–30]. The above experiment results are consistent with those in this study. The present research used corneal refractive surgery as a trauma model for the first time and proved the secretory effect of VIP. In addition, based on the immune regulation of secreted VIP on MUC5AC, the causes of postoperative MUC5AC injury can be explained in addition to intraoperative negative pressure suction [31]. The difference in VIP secretion may partly regulate the injury of MUC5AC, which requires further experimental verification.

The method used, LASEK or FS-LASIK, has different effects on dry eye symptoms post-surgery. This may be caused by the differences in fabrication of the corneal flap. In LASEK, a corneal flap is created by scraping off part of the corneal epithelium covering the ablation area. This results in disturbance and repair of the corneal environment as well as severe dry eye within 1 week, as confirmed in a rat corneal injury model [32]. In FS-LASIK, the corneal flap penetrates the corneal stroma and circularly cuts-off certain nerves, exacerbating the decrease in corneal sensitivity and blink reflexes, resulting in dry eye symptoms lasting for at least 3 months [33]. These neural injury processes reasonably explain our results. Therefore, medication for the treatment of dry eyes post-surgery can be guided by the regularity of the symptoms.

Among the factors correlated with dry eyes, subjects who underwent LASEK surgery showed positive correlation with corneal AD, but subjects who underwent FS-LASIK did not. In LASEK surgery, these results mean that the higher the AD, the higher is the possibility of dry eyes post-surgery. The degree of myopia before surgery determines corneal AD, which may explain why patients with high myopia are prone to dry eyes [8]. Therefore, for LASEK, the nerve injury caused by ablation of the corneal stroma is an important contributor to dry eye symptoms. However, for FS-LASIK, probably other reasons, such as annular transection of the nerve during corneal flap fabrication may also aggravate dry eyes. In recent years, the design of corneal flaps such as the size, shape, and position of the corneal hinge for FS-LASIK, and the popularity of small incision lenticule extraction surgery [34] have been based on this research. Therefore, dry eye symptoms after FS-LASIK surgery are a common result of corneal flap annular incision and stromal ablation.

The positive correlation between postoperative TBUT and preoperative TBUT suggests that preoperative basic ocular surface status determines postoperative dry eye syndrome, which is consistent with the results of previous clinical statistics [35]. The influence of VIP on dry eye symptoms was lesser in LASEK than in FS-LASIK, 1 week post surgery. It is speculated that other neuropeptides, such as calcitonin gene–related peptide (CGRP), may also affect the dry eye symptoms in LASEK within 1 week. Hyperalgesia and increased CGRP caused by corneal injury result in blinking and tearing, a process that maintains corneal surface homeostasis. Dry eye symptoms are accompanied by a decrease in CGRP levels after repair [32]. Therefore, in addition to VIP, CGRP probable has a protective effect in relieving dry eyes in the early stages after LASEK, which also needs further validation.

COVID-19 delayed follow-up examinations for some subjects, resulting in a reduction in the number of complete samples as well as the inability to observe and study the patients 3, 6 and 12 months post-surgery. Although the current samples were adequate for statistical analysis, further studies with a larger sample size and longer timeframes, such as 3, 6 and 12 months, would validate the accuracy of our results.

## Conclusion

In conclusion, nerve injury during surgery causes VIP stimulation and an increase in its concentration, indicating that VIP has a neuroprotective effect on postoperative DED. Based on the changes in VIP, this study reasonably explained the cause of neurogenic dry eyes after LASEK and FS-LASIK surgeries. In addition, dry eye symptoms regularity can guide clinical medication; for example, patients with dry eyes before surgery and high myopia should be given higher doses or at a higher frequency for a longer period after surgery. A certain period of high occurrence of dry eye could increase drug application to avoid complications. In future studies, the role of VIP in regulating dry eye in experimental verification of laser keratorefractive surgery will be verified again in animal models.

## Data Availability

All data generated or analysed during this study are included in this published article.

## List of abbreviations

AD: corneal ablation depth
CGRP: calcitonin gene–related peptide
DED: dry eye disease
FS-LASIK: femtosecond laser-assisted in situ keratomileusis
IOP: intraocular pressure
LASEK: Laser-assisted subepithelial keratomileusis
LASIK: laser-assisted in situ keratomileusis
OSDI: ocular surface disease index
RCT: predicted residual corneal thickness
SE: spherical equivalent
TBUT: tear-film breakup time
VIP: the vasoactive intestinal peptide.
